# Creating diverse and inclusive scientific practices for research datasets and dissemination

**DOI:** 10.1162/imag_a_00216

**Published:** 2024-07-12

**Authors:** Julia W.Y. Kam, AmanPreet Badhwar, Valentina Borghesani, Kangjoo Lee, Stephanie Noble, Pradeep Reddy Raamana, J. Tilak Ratnanather, Davynn G.H. Tan, Lena K.L. Oestreich, Hyang Woon Lee, Laura Marzetti, Hajer Nakua, Gina Rippon, Rosanna Olsen, Alyssa Pozzobon, Lucina Q. Uddin, Julio Alejandro Yanes, Athina Tzovara

**Affiliations:** Department of Psychology, Hotchkiss Brain Institute, University of Calgary, Calgary, Canada; Multiomics Investigation of Neurodegenerative Diseases (MIND) Laboratory, Département de Pharmacologie et Physiologie, Institut de Génie Biomédical, Université de Montréal and Centre de Recherche de l’Institut Universitaire de Gériatrie, Montreal, Canada; Department of Psychology, Faculty of Psychology and Educational Sciences, University of Geneva, Geneva, Switzerland; Department of Psychiatry, Yale University School of Medicine, New Haven, CT, United States; Department of Psychology, Northeastern University, Boston, MA, United States; Department of Bioengineering, Northeastern University, Boston, MA, United States; Center for Cognitive and Brain Health, Northeastern University, Boston, MA, United States; Department of Radiology, University of Pittsburgh, Pittsburgh, PA, United States; Department of Biomedical Engineering, University of Pittsburgh, Pittsburgh, PA, United States; Department of Intelligent Systems, University of Pittsburgh, Pittsburgh, PA, United States; Center for Imaging Science and Institute for Computational Medicine, Department of Biomedical Engineering, Johns Hopkins University, Baltimore, MD, United States; Department of Rehabilitation Sciences, The Hong Kong Polytechnic University, Hong Kong, China; Centre for Advanced Imaging (CAI)/Australian Institute for Bioengineering and Nanotechnology (AIBN) and School of Psychology, The University of Queensland, Brisbane, Australia; Department of Neurology, Ewha Womans University School of Medicine, Seoul, South Korea; Department of Medical Science, Ewha Womans University School of Medicine, Seoul, South Korea; Computational Medicine, System Health Science and Engineering, and Artificial Intelligence Convergence Graduate Programs, Ewha Womans University, Seoul, South Korea; Department of Neuroscience, Imaging and Clinical Sciences, University of Chieti-Pescara, Chieti, Italy; Institute for Advanced Biomedical Technologies, University of Chieti-Pescara, Chieti, Italy; Centre for Addiction and Mental Health, Institute of Medical Science, University of Toronto, Toronto, Canada; Institute of Health and NeuroDevelopment, Aston University, Birmingham, United Kingdom; Rotman Research Institute, Baycrest Academy for Research and Education, Toronto, Canada; Department of Psychology, University of Toronto, Toronto, Canada; Department of Psychology, Faculty of Social Science, University of Ottawa, Ottawa, Canada; Department of Psychiatry and Biobehavioral Sciences, University of California Los Angeles, Los Angeles, CA, United States; National Center for Complementary and Integrative Health, National Institutes of Health, Bethesda, Maryland; Institute of Computer Science, University of Bern, Bern, Switzerland; Center for Experimental Neurology, Sleep Wake Epilepsy Center, NeuroTec, Department of Neurology, Inselspital, Bern University Hospital, University of Bern, Bern, Switzerland

**Keywords:** diversity, equity, inclusivity, artificial intelligence, research barriers, OHBM

## Abstract

Diversity, equity, and inclusivity (DEI) are important for scientific innovation and progress. This widespread recognition has resulted in numerous initiatives for enhancing DEI in recent years. Although progress has been made to address gender and racial disparities, there remain biases that limit the opportunities for historically under-represented researchers to succeed in academia. As members of the Organization for Human Brain Mapping (OHBM) Diversity and Inclusivity Committee (DIC), we identified the most challenging and imminent obstacles toward improving DEI practices in the broader neuroimaging field. These obstacles include the lack of diversity in and accessibility to publicly available datasets, barriers in research dissemination, and/or barriers related to equitable career advancements. In order to increase diversity and promote equity and inclusivity in our scientific endeavors, we suggest potential solutions that are practical and actionable to overcome these barriers. We emphasize the importance of the enduring and unwavering commitment required to advance DEI initiatives consistently. By doing so, the OHBM and perhaps other neuroscience communities will strive toward a future that is not only marked by scientific excellence but also characterized by diverse, inclusive, and equitable opportunities for all, including historically under-represented individuals around the world.

## 1 Introduction

Diversity, equity, and inclusivity (DEI) initiatives are critical for scientific innovation and progress. Most academic institutions and societies have by now formed committees that are dedicated to pursuing DEI initiatives. In the “early” days, DEI initiatives focused mainly on addressing gender disparities by increasing the proportion of women in visible positions, such as conference organizers and editorial board members. While these efforts have increased opportunities for women scientists in recent years, additional effort and progress are required to ensure that women and other historically under-represented groups are adequately represented as prominent researchers and leaders within the field. The major remaining barriers include, but are not limited to, representation at international conferences and participation in research panels, publications in “prestigious” journals, and the number of citations of papers led by women and other historically under-represented groups (Chatterjee & Werner, 2021;Liu et al., 2023;Llorens et al., 2021). The Organization for Human Brain Mapping (OHBM) recognized these imminent needs and formed the Diversity and Inclusivity Committee (DIC) in 2016, with the goal of promoting diversity, equity, and inclusivity within the international brain mapping community.

The primary goal of this article is to highlight three ongoing obstacles and to identify both opportunities and solutions to overcome these obstacles, in order to increase diversity and promote equity and inclusivity across all scientific endeavors. These obstacles include lack of diversity and accessibility to publicly available datasets, language barriers in research dissemination, and barriers related to equitable career advancement. Expanding beyond our previous work that highlighted the major DEI initiatives undertaken by OHBM to promote diversity and inclusivity in our community (Tzovara et al., 2021), here we highlight some of the current pressing barriers to DEI progress along two main axes: (a) within human-centered brain research practices and (b) research dissemination practices. The first obstacle corresponds to the first axis, and includes the lack of diversity in and accessibility to publicly available datasets, which limits the generalizability and scope of research output. The second and third obstacle corresponds to the second axis, which concern language barriers in research dissemination and barriers in equitable career advancements. These obstacles create inequalities and can directly limit the career development of researchers from historically under-represented groups and countries in science. A graphical summary of these obstacles and potential solutions are illustrated inFigure 1. We recognize that these obstacles are not the only ones that need to be overcome, and others with different lived experiences may identify different challenges as more pressing. Finally, we draw upon our experiences as members of the OHBM DIC and lessons learned from existing literature to offer practical and feasible solutions when possible.

**Fig. 1.**
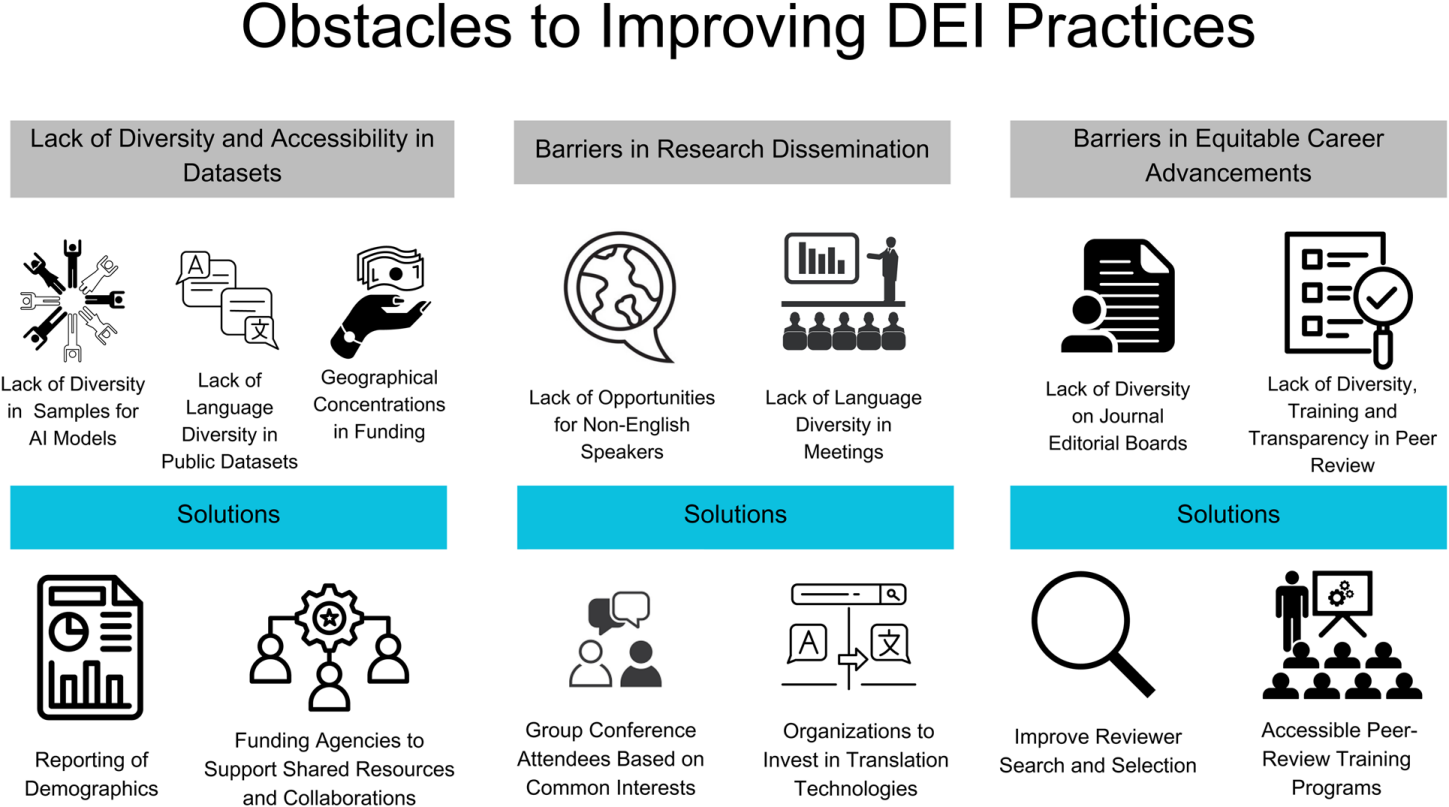
Graphical summary of obstacles and potential solutions to diverse and inclusive practices for research datasets and dissemination.

## 2 Lack of Diversity in and Accessibility to Publicly Available Datasets

### 2.1 Lack of diversity in publicly available datasets

In the field of neuroimaging, high-quality data are an indispensable part of the research process. They allow researchers to test hypotheses about the brain and mind, fit models, draw conclusions, and generate new theories. In recent years, computational and artificial intelligence (AI) techniques have become increasingly popular and represent a powerful tool for data analysis of complex datasets, particularly human neuroimaging data. In parallel, there has been a remarkable increase in initiatives to gather large datasets to study the human brain using powerful computational methods. With increased attention to inclusive approaches to precision medicine and to improve clinical translation and fair outcomes, the neuroimaging community has become aware of the need to improve recruitment efforts from diverse groups. Improving the diversity of participants necessitates the consideration of race/ethnicity, sex, and age in brain research, particularly as they pertain to drug development, clinical decision making, and mental health outcomes to ensure adequate representation of different populations and to improve generalizability of research outcomes (Kopal et al., 2023;Li et al., 2022). Although incidence rates, prevalence, and risks of neurological and psychiatric disorders vary between different racial/ethnic groups, most health research have focused on studying predominantly White, college-educated populations from North America and European countries (Babulal et al., 2019;Haga, 2010;Lim et al., 2023;Mills & Rahal, 2019;Velarde et al., 2023).

Moreover, reporting demographic information is not required by most neuroimaging journals and is poorly documented in most publications (Goldfarb & Brown, 2022;Li et al., 2022;Sterling et al., 2022;Tzovaras & Tzovara, 2019). Neuroimaging studies that have reported demographic information have revealed an over-representation of samples from W.E.I.R.D. (Western, Educated, Industrialized, Rich and Democratic) societies (Henrich et al., 2010). This lack of reported information about the demographic information of the studied research sample limits the ability to draw conclusions about the generalizability of the findings. One example of the limitations in understanding human cognition when primarily using WEIRD samples lies in the neurobiology of language. Within this field, there is an Anglocentric bias (i.e., the vast majority of studies have centered on monolingual native English-speaking populations), and a first-language bias (i.e., current available instruments are meant to be administered in the participant’s native language). Yet, there are 7,139 languages spoken worldwide (Ethnologue, 2022); within the United States, 22% of the households are bilingual (Dietrich & Hernandez, 2022). We return to this language issue in a later section.

It has also been suggested that ancestral, geographical, and environmental diversity in study participants should be considered particularly in genome-wide association studies (GWAS) (Fitipaldi & Franks, 2023) on Alzheimer’s disease (Dehghani et al., 2021), dementia (Sirkis et al., 2019), and Parkinson’s disease (Bandres-Ciga et al., 2020). Yet, the reporting of demographic information remains limited in the majority of studies. Without adequate reporting of demographic information (particularly race/ethnicity data), it is difficult to know which research findings have the potential to be generalizable to various populations and which do not.

The issues of understudied populations and lack of participant diversity in large open datasets introduce bias in brain research and clinical trials. For example, studies using AI techniques can be significantly impacted by biases in participant recruitment or selection, and used algorithms (Chen et al., 2021;Denton et al., 2020;Norori et al., 2021;Parikh et al., 2019). The application of AI to neuroimaging data often aims to train models using a large amount of training data and perform prediction of research/clinical targets (e.g., tumor segmentation, disease risk, disease progress, treatment outcome) on an independent test dataset (e.g., from newly admitted patients) (Arlot & Celisse, 2010;Choi & Sunwoo, 2022). The disparity of available data for certain groups may lead to discrepancies between training and test datasets, which may result in biased or unsatisfactory outcomes due to dataset shifts that are limited in its generalizability (Chekroud et al., 2024;Park et al., 2021;Quiñonero-Candela, 2009). For instance, when training AI algorithms on neuroimaging data collected from primarily White Americans, out-of-sample prediction errors were higher for African Americans than for White Americans (Li et al., 2022). A related issue concerns MRI templates and atlases that are often created from Western (primarily White) participants. This may lead to more biases around volumetric or functional derived metrics for non-White or non-Western participants. Indeed, studies have reported notable differences in brain morphology across ethnic and racial groups (Xie et al., 2015) and that the use of mismatched templates in spatial normalization led to substantial changes in segmentation and registration accuracy (Yang et al., 2020). Notably, all these challenges noted here not only apply to publicly available datasets but also extend to all datasets and may, therefore, limit the generalizability of some scientific findings.

Efforts to improve diverse brain research can be accomplished across multiple levels of the research ecosystem (from researchers, journal editors, and funding agencies) and across multiple research steps (from study design, experimental materials, data collection, data analysis, interpretation to replication in independent datasets) (Garcini et al., 2022;Ricard et al., 2023). We offer potential solutions to address the lack of diversity in neuroimaging datasets at the following steps: participant recruitment, experimental materials, data analysis, and/or interpretation.

In terms of participant recruitment, the lack of diversity in research participants has negative repercussions not only in understanding fundamental human cognition (Blasi et al., 2022) but also in efforts in enhancing generalizability as well as clinical and translational utility (Garcia et al., 2023). In addition to ensuring diversity in age and sex/gender, other aspects such as inclusion of individuals with disabilities or sensory impairments also need to be considered. While including such individuals has traditionally been challenging and rare due to most scanning environments lacking the proper set-up needed to accommodate their needs, current technological advances may provide solutions. For example, a potential solution to including individuals with hearing loss would be to use auto-captions so that information can be relayed by the experimenter. Another characteristic to consider lies in the spoken languages of participants. For instance, to elucidate the universal neural correlates of language processing, neurolinguistics would need to expand beyond English, and embrace and study the rich diversity offered by typologically distinct languages. Such a paradigm shift is critical given that multilinguals represent more than half of the world’s population (Grosjean, 2010), suggesting that focusing on English-only samples will limit generalizable conclusions around the neurobiology of language.

In addition, scientific journals and grant agencies have the potential to play a prominent role in increasing diversity within research recruitment efforts among research samples. For instance, if journals enforce policies in which they require the reporting of demographic information beyond age and gender, more researchers will be incentivized to include these data leading to increased understanding of the potential generalizability of a given result, so that diversity in the study sample is explicitly acknowledged. Importantly, the information provided should be aggregated so as to not compromise on the privacy of participants. Such reports should naturally consider questions of privacy and remain at an aggregate level, as providing publicly detailed reports on the demographic characteristics of each participant may compromise their right to privacy. Another solution is for funding agencies to require grant proposals to state a clear plan for achieving diversity in participant recruitment and a justification for why researchers decided to focus on a specific group. For example, the National Institutes of Health, a major funding agency in the United States, requests race and ethnic information for grant applications and progress reports, as well as mandates equal examination of biological male and female human (and animal) subjects. Similarly, the Canadian Institutes of Health Research have implemented a requirement of applicants to describe their plan for recruiting a diverse sample and to implement sex- and/or gender-based analysis when applicable. Such requirements will necessarily be country specific to ensure that datasets are representative of the diversity of that country. Notably, countries do differ in their ethnic racial diversity so it is important to consider the demographic makeup of a given country in order to recruit a representative sample. To that end, grant review committees should evaluate whether any population-specific analysis is necessary for the proposals and recommend researchers to acquire, share, and use diverse open-source databases.

In terms of experimental materials, there are several opportunities to enhance diversity in publicly available datasets. The main challenge in achieving this goal involves language barriers. Most self-report questionnaires in cognitive test batteries are written in English, limiting subject recruitment to English-speaking populations. Some behavioral tests (e.g., neuropsychological) can be intrinsically biased due to cultural differences with the tests being validated in Western, and mainly, WEIRD samples. Moreover, several commonly used tasks in functional magnetic resonance imaging (fMRI) studies may require English proficiency, such as when participants are required to listen to narratives or respond to questions, and may implicitly require an understanding of Western culture (Finn et al., 2018;Youssofzadeh et al., 2022). One possibility is for research groups to consider developing novel experimental protocols that do not require language skills unless strictly needed (e.g., when studying language). Developing broader experimental protocols that rely on images, sounds, or symbols can also have the additional benefit of improving the representation of participants recruited among those who are not fully literate in English, broadening even more the generalizability of experimental findings.

In terms of data analysis and interpretation, one solution is to train predictive models based on more diverse samples. A recent study found training functional connectivity-based predictive models on more diverse data improved predictions in some cases (Adkinson et al., 2024). Diversifying research samples may be better addressed by scientists in countries with a multiethnic population or through multicountry collaborations. There have also been efforts to develop templates for different ethnic groups (Xie et al., 2015). Given the existence and continuing development of anatomical templates for non-Western populations, researchers in these non-Western countries could consider using these population-specific templates for analyzing the data of their non-Western research participants. This may be particularly suitable for scientists who work in countries with a less diverse ethnic makeup (e.g., Japan and Korea). In addition, novel analysis procedures are also necessary to effectively deal with increases in brain diversity or variance introduced due to neuroplastic processes experienced by individuals with disabilities or sensory impairments. Notably, datasets that are diverse or highly specialized are beneficial in their own ways, as long as data are interoperable: the former can ensure that an algorithm has been exposed to a wide range of samples and is generalizable, while the latter can be used to fine tune existing algorithms to local populations, for example, via federated learning approaches (Rieke et al., 2020).

Securing the financial sustainability of diversity and inclusion initiatives in scientific research, particularly for datasets and dissemination, necessitates a multifaceted funding strategy. A key source includes grants from research funding bodies that prioritize diversity. As governments worldwide begin to recognize the importance and value of including minority groups in research, such funding has become increasingly available. Initiatives such as the diversity, equity, inclusion, and accessibility (DEIA) framework by the National Institutes of Health (NIH) in the United States and the National Health and Medical Research Council’s (NHMRC) emphasis on consumer involvement and prioritization of priority populations in Australia aim to ensure research is conducted in a more inclusive and diverse manner. Partnerships with industry stakeholders who recognize the value of inclusive research, along with institutional support from academic entities (Britez Ferrante et al., 2024), are also crucial for funding diverse and inclusive research efforts. For instance, industry partners and academic institutions may offer to match or supplement funding provided by government bodies to ensure diverse recruitment strategies and open access policies. Individual organizations or philanthropic foundations often provide smaller amounts of fundings for targeted research projects, including those involving populations commonly neglected or overlooked in research (Doykos et al., 2016). While these grants may not support large-scale projects, they are invaluable for conducting pilot studies, which can then bolster larger funding applications. Moreover, by engaging the broader community and demonstrating the impact of inclusive research practices, projects can attract donations from individuals and organizations committed to diversity and inclusion. Finally, implementing cost-saving measures, such as utilizing open-source tools for data analysis and dissemination, can help reallocate funds toward diversity and inclusion efforts. Together, these strategies can provide a robust financial foundation to support and sustain diversity and inclusion efforts in the scientific community, ensuring that research practices and dissemination are accessible and representative of diverse perspectives.

### 2.2 Limited access to publicly available data

In addition to the lack of diversity in participants included in public datasets, there are also barriers around accessing publicly available datasets for many under-represented researchers. These barriers are likely due to (1) a lack of basic infrastructure, including network availability and access to a reliable power grid; (2) lack of resources for storing the data and implementing the analyses; (3) requirements of affiliations with partnering institutions; (4) requirements of an application to access the data (which may discourage some researchers); and (5) access fees for the transfer of large datasets, even if the use of the data itself is free. In the human neuroimaging community, large-scale data collection and sharing initiatives such as the Human Connectome Project (HCP) (Van Essen et al., 2013), the U.K. biobank (Sudlow et al., 2015), and Adolescent Brain Cognitive Development (ABCD) (Casey et al., 2018) have made large datasets available to researchers. However, accessing and managing them (e.g., to download, host, and process these data) require expertise in research data management as well as access to large storage servers and high-performance computing systems. For example, raw data from the ABCD dataset comprise 1.35GB per individual totaling to about 13.5TB for the first release of over 10,000 individuals (Horien et al., 2022). The computational resources necessary to handle such data can be challenging to obtain for early career researchers and those working in institutions that do not support supercomputing facilities. While cloud computing services are being increasingly utilized to process neuroimaging data and some services do not incur costs for its users (brainlife.io;Hayashi et al., 2024), the use of these services can come at a fairly significant cost, which poses another potential barrier for under-funded research groups. This necessitates novel initiatives aiming at increasing access to data analysis ecosystems, for instance via virtual desktops (Renton et al., 2024). Taken together, these resource barriers prevent some researchers (often those from marginalized groups and from countries or institutes with limited budgets for research) from accessing publicly available datasets that are intended for the global scientific community.

Accessibility to publicly available datasets and computational resources can be enhanced in several ways. Solutions for removing resource barriers to enhance accessibility to publicly available datasets require efforts that are broadly relevant for addressing other barriers. For example, research institutions offering funds for supporting supercomputing facilities may be a promising solution especially for early career researchers. Yet, in-house supercomputing facilities are not necessarily provided by many research institutes around the world. Such facilities are extremely costly and may require specialized personnel, including experts in cybersecurity, and infrastructure that is resilient to natural disasters or power failures, and would require financial security in the long term, which is not always guaranteed. These considerations are already starting to result in a divide in computational capacities between academia and industry for demanding computation, such as powerful AI models, the existing computational capacities may already be insufficient in academia. Having such facilities in house is far from being a reality in many institutes, which would then need to consider alternative cloud-based resources for data storage and computations. As these services are also costly, the modern needs for computational power are prone to result in further inequalities in the upcoming years. In particular, beyond the U.S. and U.K. dominance in funding sources of research (e.g., National Institute of Health and Wellcome Trust;Mills & Rahal, 2019) is the socioeconomic disparity between the Global North and South (Mammen et al., 2023). This disparity limits access to education and technology (which is particularly relevant in neuroscience), and may thereby present as challenges to current diversity, equity, and inclusion initiatives. We recognize that there is no straightforward solution that is ready to be implemented and raise the need for future deliberation on how to overcome such inequalities.

One important consideration related to available financial resources is the carbon footprint associated with such supercomputing facilities, which require high levels of energy consumption and, therefore, incurs high energy costs (Bates et al., 2015). Recent efforts have been made to successfully increase energy efficiency at a supercomputing facility in the United Kingdom which also reduces the energy cost (Jackson et al., 2023). This initiative needs to be more widely implemented across supercomputing facilities in order to collectively reduce their impact on the environment. Another potential solution would be for funding agencies to support collaborations between researchers across countries (particularly between high- and low-resource countries) to allow for resources to be shared. International scientific organizations such as OHBM also have an active role to play in this regard, by facilitating scientific exchanges and collaborations among researchers for sharing resources as well as supporting the efforts of neuroscientists in the Global South by providing financial and other types of resources to the Latin American Brain Mapping Network (https://www.ohbmbrainmappingblog.com/blog/the-language-of-ohbm-is-universal). Another way in which scientific societies can contribute is by providing additional member benefits for researchers working in under-resourced settings. For example, OHBM recently launched a “Membership+” initiative that provides unlimited space on Dropbox for members (Steinkamp, 2023). OHBM also has launched an open-source publishing platform, Aperture Neuro, with a reduced publishing fee.

Supercomputing facilities and services such as Amazon Web Services may also consider allocating pro bono computing time for researchers without means to financially compensate them. Notably, the ability to obtain funding is closely associated with other barriers described here, which we elaborate in other sections.

## 3 Language Barriers in Research Dissemination

English has become the lingua franca of science (Debat & Abdill, 2020), such that avenues to communicate science (such as journal publications, presentations, grants) occur almost exclusively in English. Scientific progress relies on the diverse transfer of ideas to fuel theories, methods, and implementations (Hong & Page, 2004). The historical dominance of the English language in science has excluded much of the scientific world in which languages other than English (LOTE) are the primary language from contributing to and learning from scientific advances in the Western world. If the English language continues to completely dominate the scientific landscape, then there is an imposed selection bias on the ideas being shared and transferred in science, thus limiting diverse and novel ideas from improving scientific discovery.

The dominance of English in science presents several challenges to inclusive scientific research (Amano et al., 2023;Blasi et al., 2022). In particular, writers of LOTE face barriers in disseminating their research in English through publications or presentations due to their difficulty with English proficiency (Amano et al., 2023). Specific to publications, writers of LOTE have to incur additional costs to engage translation and/or editing services, and often face frequent rejection or revision due to reasons related to grammar or English writing style (Ramírez-Castañeda, 2020). Unfortunately, the ethnicity or country of origin of the authors rather than the grammar per se may influence the evaluation of the writing (Lindsey & Crusan, 2011). It has been suggested that a potential solution is that journals or other resources could help absorb the cost of editing and translation services if the article contributes to the scientific community (Khelifa et al., 2022;Ramírez-Castañeda, 2020;Steigerwald et al., 2022). In terms of enhancing the quality of writing, native English speakers within large-scale collaborations may help with editing the papers written by speakers of LOTE. Reviewers are encouraged to comment constructively to guide the use of English rather than give generic comments on the lack of English mastery (Romero-Olivares, 2021). Publishers can also help by adding a section consisting of suggestions on language use in Guidelines to Authors (Tychinin & Webb, 2003). Moreover, journals could require reviewers to indicate which LOTE they use. In this way, an article could first be reviewed in the authors’ native language and, if accepted, be translated by a professional translation service to English. In terms of translation services, an effective example is DeepL, which is a tool powered by artificial intelligence to translate text in documents and webpages while preserving nuances in the original content.

Specific to conferences, speakers of LOTE may not be able to articulate their ideas as proficiently in English as in their native languages although they can communicate in English generally. Differences in nonverbal body language and linguistic differences across cultures present an additional barrier, as speakers of LOTE may find it difficult to pick up on verbal and nonverbal cues important for understanding the context of presentations or scientific discussions. In addition, language and cultural barriers can impact social networking of speakers of LOTE in social events during conferences. For example, while the main objectives of social events at OHBM are to promote interactions among attendees with different backgrounds and affiliations with or without a specific agenda (e.g., welcome/evening receptions or special interest groups), networking in a second or third language is a difficult task for many LOTE attendees from outside of U.S./European-centralized institutional networks. Topics covered in social conversations often go beyond science, where cultural differences also impact the understanding and engagement of speakers of LOTE.

One potential solution to overcome these language/cultural barriers is to have a minimal event structure for introducing and grouping attendees based on common interests that can help guide attendees to form conversation circles without any prior social connections. Moreover, it has been suggested that senior and established scientists can take a more active and intentional role in informally or casually mentoring early career researchers from historically under-represented groups (Uddin & De Los Reyes, 2021). One way to do this would be for senior mentors to advertise via social media or through the conference organizers a willingness to act as a point of contact at a meeting, or offer to introduce early career researchers to a particular individual or network of individuals. Networking can be particularly challenging for those who are speakers of LOTE, which may lead to missed opportunities for casual conversations that occur at social events. Having points of contact may thus facilitate participation in social activities. Another alternative is for conferences to allow individuals to self-organize groups of interest in a grass-roots approach, which can also increase opportunities for early career researchers and individuals who are comfortable with LOTE to socialize and expand their networks.

Technical solutions to language barriers during conference presentations are also beginning to emerge. While it is now possible to provide auto-captions (which is distinct from closed captions) via several presentation software (e.g., Microsoft Powerpoint 365 or Google Slides), it is not clear whether accurate translation to the native language of the user can be achieved in this way. Fortunately, it is possible to use sound mixer software to integrate audio into translation apps such as Microsoft Translator or Google Live Transcribe. In conference mode with a five letter keycode, Microsoft Translator provides auto-captions in the desired language. Conversely, the speaker may choose to speak in their native language and provide a keycode for others to get the auto-captions. Three crucial factors will determine positive outcomes of these services: (i) clear speech, (ii) use of high-fidelity microphones, and (iii) good internet or wifi speed. With clear speech and high-fidelity microphones, the word error rate for technical content in English can be as low as 2–3% (Cooke et al., 2020) and for human captions is as low as 5%. The word error rate for technical content in different languages remains unknown. With Automatic Speech Recognition (ASR) apps, the word error rate was as low as 5-15% with training (Xiong et al., 2018). Notably, ASR apps tend to perform optimally with American accents in English, which is disadvantageous for English speakers with other accents and speakers of LOTE. Although such ASR apps (such aswww.worldly.ai) are being increasingly used at conferences, diversity of accents and languages in training the artificial intelligence is necessary for this tool to be maximally effective. Moreover, speakers of LOTE could benefit from auto-captions customized for their speech (Biadsy et al., 2019;Doshi et al., 2021) via the Project Relate app (https://sites.research.google/relate/). A related issue concerns those who rely on hearing aids to interact with others at conferences. Many hearing aids do not effectively distinguish speech from noise, and instead amplify all sounds, which make it difficult to comprehend conversations at conferences and social gatherings. Finally, an overall by-product of captions for everyone including those who have auditory processing issues is improved comprehension and reduced stress (Tzovara et al., 2021).

## 4 Barriers Related to Equitable Career Advancement

Although recent years have witnessed increased efforts in acknowledging gender and racial disparity in publications and citations (Carr et al., 2017;Llorens et al., 2021;Schrouff et al., 2019), barriers related to publishing continue to disproportionately impact under-represented researchers. For example, articles that are published by a White researcher (first or last author) get cited more than articles authored by a non-White researcher (Bertolero et al., 2020;Liu et al., 2023). Given that publication and citation of papers are among the most widely used metrics of success in academia, impacting hiring decisions, tenure prospects, and grant success among others, ensuring equity in publication and citation practices is an important step toward enabling scientists of all backgrounds to advance in their academic careers.

Publication barriers are closely linked to the aforementioned challenges, however, some of the most directly relevant barriers are in the hands of editorial boards. At most journals, editors hold substantial power over which papers are reviewed and ultimately published, directly impacting the academic success of authors. There is currently a lack of geographical and ethnic diversity on editorial boards of many scientific journals, particularly among higher impact journals (Altman & Cohen, 2021;Liu et al., 2023;Palser et al., 2022), with the predominantly represented country being the United States. This is particularly problematic given the strong association between the nationality of editorial board members and authors from those journals (García-Carpintero et al., 2010), though the directionality of this relationship is undetermined. One potential solution is to diversify editorial board members. A step toward that target involves increasing awareness and transparency in the demographic make-up of editorial boards. For example, the journal*Neuroscience and Biobehavioral Reviews*reports the breakdown of geographical location of their editorial board. Another potential solution involves the journal or the editor ensuring that both reviewers and cited authors are representative of the base rate in the field. For instance, in recruiting more women reviewers in neuroscience, the Nature Publishing Group has reached a gender ratio that aligns with the base rate in the field. Journals can also require a citation diversity statement from authors that describes the diversity of their referenced papers, with the ultimate aim to mitigate citation bias by raising awareness of this issue in our own work (Zurn et al., 2020). Another promising solution is to consider diversity in citations (Fulvio et al., 2021). For instance, the*Journal of Cognitive Neuroscience*encourages authors to increase diversity in the citations and to report their articles’ gender citation balance (JoCN Submission Guidelines, n.d.). Although this currently includes a breakdown of citations in terms of inferred male/female authors, future extensions can also include a citation diversity statement that also considers other aspects of diversity, such as geographic diversity (Zurn et al., 2020).

Similar barriers can also be found within the peer review process. The scientific community largely agrees that peer review represents an essential component of academic research (de la Garza & Vashi, 2022;Mulligan et al., 2013). Yet, numerous barriers related to peer review limit equitable opportunities for historically under-represented groups (Hall et al., 2019). One potential solution involves peer review training programs. Existing efforts to improve the peer review process have centered around providing guidance to reviewers about constructing professional and thoughtful reviews (Aly et al., 2023;Blockeel et al., 2017;Ford, 2019), creating resources to enhance and/or assess peer review caliber (Hall et al., 2019;Thompson et al., 2016), or proposing peer review training programs (Chong, 2021;Newcomb & Lockey, 2021). Such programs are warranted, given that scientists rarely receive formal training in peer review best practices, and unprofessional peer reviews have been shown to disproportionately harm under-represented groups (Silbiger & Stubler, 2019). Critically, however, these training programs are also not readily accessible (Willis et al., 2023) and evidence regarding their effectiveness has been mixed (Bruce et al., 2016;Callaham & Tercier, 2007;Doran et al., 2014;Galipeau et al., 2015), which underscores the need for focused, evidence-based initiatives to improve diversity in peer review (Willis et al., 2023). Other efforts have centered around improving transparency in the peer review process, for example, through “open” (i.e., unblinded) review. For example, the peer review model at eLife involves a collaborative process between editors and reviewers, in which the joint assessment of the manuscript is then published online (https://elifesciences.org/peer-review-process). Yet, the nontransparent selection of manuscripts to be selected for this new model of publications has raised some concerns from the community, which suggests that there is still work to do to identify new models for transparent peer review. Despite increasing support for open review among academic researchers (Delikoura & Kouis, 2021;Wolfram et al., 2020), less than 10% of academic journals in ecology, economics, medicine, physics, and psychology offer some form of open review (Hamilton et al., 2020). Although open reviews improve professionalism in peer reviews, care should be taken to ensure that possible gender, racial/ethnic, and/or geographic biases are not inadvertently introduced into the process (Souder, 2011). Another solution for reducing selection bias within the peer review process at the level of journals involves the use of improved reviewer search tools as part of the editorial management systems. This involves systematically searching for reviewers based on content keywords using these tools instead of relying on the editor’s network or reference list. Finally, other, innovative efforts to improve peer review include (1) collaborative review between authors, reviewers, and editors to reach consensus regarding manuscript content, (2) prepublication community review involving the use of preprint servers, and (3) outsourcing reviews to third-party service providers (for a review, seeBarroga, 2020). Importantly, a growing trend among academic researchers involves increasing reluctance to participate in peer review which stems from several reasons (e.g., lack of compensation, time constraints): reasons which have likely been amplified during the COVID-19 pandemic (Kerig, 2021). This puts a strain on remaining reviewers; yet, peer reviewed publication is central to academic career progression. Therefore, future work needs to focus on evidence-based initiatives that improve diversity throughout the peer review process.

Finally, in addition to barriers related to publishing, similar challenges that disproportionately impact historically under-represented researchers have been observed in other domains that are critical for career advancement. Symposium presentation at professional meetings is a notable marker of success in academia, for both the symposium organizer and the invited presenters, yet gender bias in this domain is pervasive. For example, women are less likely than men to be speakers at conferences (Schroeder et al., 2013;Schrouff et al., 2019). Similarly, awards are a critical metric of success, and under-represented researchers are disproportionally impacted. For instance, women are less likely to receive prestigious awards (such as young investigator awards and Nobel prizes;Lunnemann et al., 2019;Melnikoff & Valian, 2019). A potential solution to these barriers is that evaluation committees for conference presentations and awards take a more holistic approach in their assessments of researchers’ scientific work. Specifically, this involves stepping away from solely relying on the number of publications or citation indices and considering research impact in the context of available resources as well as the impact of other research activities in addition to publishing (e.g., sharing research tools). A complete list of such challenges goes beyond the scope of this work and has been reported elsewhere (Llorens et al., 2021), highlighting the need for developing concrete solutions. In addition to gender bias, other biases in relation to ethnicity, disabilities, sexual orientation, or religion, to mention a few, also persist across many domains in academia. Future initiatives need to consider biases that involve intersecting identities in order to enhance equity across all academic activities.

## 5 Conclusion

Recent years have witnessed increasing number of DEI initiatives across academic institutions and societies. While these initiatives may have improved opportunities for historically under-represented scientists, multiple barriers continue to prevent them from achieving equitable career trajectories as researchers from Western countries. As members of the OHBM Diversity and Inclusivity Committee, we presented our experience and lessons from the existing literature to describe three main barriers limiting DEI practices in the neuroimaging community, as well as offer potential solutions to overcoming these barriers. The ultimate goal is to increase diversity and promote equity and inclusivity in scientific communities and research practices. Additional considerations beyond the three barriers described in this paper are important to note; for example, as online conferences become increasingly common, intentional efforts are needed to ensure that conferences are designed to encourage the meaningful participation of participants from under-represented groups (Gau et al., 2021;Levitis et al., 2021). Moreover, the carbon footprint associated with certain DEI initiatives, such as the proposition to host conferences in diverse locations for broader accessibility, is also a pivotal concern. Striking a delicate balance between advancing these inclusive initiatives and adhering to environmental and sustainability principles poses a complex dilemma that warrants careful consideration. Although much work still needs to be done by individuals at all levels of the research ecosystem, we are optimistic that our goals for improving diversity, equity, and inclusivity among scientific communities can be realized through a collective effort to implement future evidence-based initiatives.

## Data Availability

Data sharing is not applicable to this article as no new data were created or analyzed in this study.
